# Peritonitis caused by *Ewingella americana* in a patient with peritoneal dialysis: a case report

**DOI:** 10.1186/1752-1947-8-86

**Published:** 2014-03-04

**Authors:** Li Li, Jianxiong Shen, Jianmin Tao, Zhizhong Xue

**Affiliations:** 1 Yueyang Hospital of Integrated Traditional Chinese and Western Medicine, Shanghai University of Traditional Chinese Medicine, No.110, Ganhe road, Shanghai 200437, China

**Keywords:** Peritonitis, Peritoneal dialysis, *Ewingella americana*

## Abstract

**Introduction:**

Peritonitis can happen in patients undergoing continuous ambulatory peritoneal dialysis. It is commonly caused by *Staphylococcus aureus,* which is the basis of the empiric choice of antibiotics. Here we report a case that was caused by *Ewingella americana* and thus required different considerations of treatment.

**Case presentation:**

A 76-year-old Chinese woman underwent continuous ambulatory peritoneal dialysis after the diagnosis of chronic renal failure. She presented with generalized abdominal pain after the start of continuous ambulatory peritoneal dialysis for 10 consecutive months. The pathogen was identified as *Ewingella americana* by microbiological examination and its susceptibility to antibiotics was partly different from the common pathogens causing peritonitis. The change of antibiotic treatment from vancomycin to amikacin accelerated her recovery.

**Conclusion:**

It should be noted that peritonitis can be caused by *E. americana.* Our report may add to the known causes of peritonitis in a hospital environment. With respect to the empiric choice of antibiotics to treat peritonitis under the condition of continuous ambulatory peritoneal dialysis, it is worth noting that *E. americana* has a different susceptibility to the spectrum of antibiotics and its corresponding therapy should be taken into consideration.

## Introduction

Peritonitis is mainly caused by infection due to the disruption of the peritoneum, commonly under the condition of continuous ambulatory peritoneal dialysis (CAPD). Peritoneal dialysis (PD) is used as a treatment for patients with severe chronic renal disease. The process uses the patient’s peritoneum in the abdomen as a membrane across which fluids and dissolved substances (electrolytes, urea, glucose, albumin and other small molecules) are exchanged from the blood. This regular exchange is performed by catheterization in the abdomen which increases the opportunity for infection. Peritonitis primarily caused by bacteria has more clear symptoms of infection in contrast to those subsequent to perforation of part of the gastrointestinal tract.

*Ewingella americana* was first described from clinical samples by Grimont *et al.* in 1983 [1]. It rarely causes human infections, although several investigations reported its existence in sputum [2], conjunctiva [3,4], blood [5-8], wounds [9], peritoneal dialysis [10] and bone marrow [11].

Here we report a case of peritonitis due to *E. americana* in a patient with end-stage renal disease undergoing CAPD.

## Case presentation

A 76-year-old Chinese woman underwent CAPD after the diagnosis of chronic renal failure (uremia). Surgery for peritoneal dialysis was performed by successful catheterization under the help of local anesthesia. She was admitted due to generalized abdominal pain after a period of 10 months of CAPD. She had symptoms of decreased appetite, left chest pain and pressure, which was worse at night, but no nausea and acid regurgitation. Physical examination revealed tenderness and positive rebound.

The hematological tests revealed a hemoglobin level of 10.9g/dl, a platelet count of 201×10^9^/liter, a white cell count of 11.6×10^9^/liter. The peritoneal dialysate was turbid, and microscopic examination showed 400 cells/mm^3^, with a predominance of neutrophils. The diagnosis of peritonitis was established, and our patient was treated empirically with vancomycin intravenously. Samples of dialysate were obtained and inoculated onto 5% sheep blood agar. After 48h of incubation at 37°C, a gram-negative, lactose-fermenting rod was observed that showed oxidase negative and catalase positive. The isolate was identified by the VITEK® compact identification systems (bioMérieux Vitek Inc., Hazelwood, MO, USA) as *E. americana. E. americana* was further tested by sequencing the 16s ribosomal deoxyribonucleic acid (rDNA) (Sangon Biotech, Shanghai, China) and confirmed by comparison with the genetic sequence published previously.

Antimicrobial susceptibility was determined by the VITEK® compact system (using card AST-GN 13). The isolate was found to be susceptible to tobramycin, ceftazidime, cefepime, aztreonam, imipenem, amikacin, gentamicin, ciprofloxacin, piperacillin and/or tazobactam and levofloxacin but resistant to ampicillin, ampicillin and/or sulbactam, cefazolin, cefotetan, ertapenem, trimethoprim and/or sulfamethoxazole and nitrofurantoin (Table 1). After susceptibility results were obtained, vancomycin therapy was stopped and changed to amikacin until our patient’s complete recovery.

**Table 1:** **Antimicrobial susceptibility testing of *E. Americana***

**Antimicrobial agent**	**MIC (μg/ml)**	**Interpretation**
Ampicillin	>=32	R
Ampicillin and/or Sulbactam	>=33	R
Cefazolin	>=64	R
Cefotetan	>=64	R
Ertapenem	>=8	R
Tobramycin	<=1	S
Trimethoprim and/or Sulfamethoxazole	80	R
Ceftazidime	<=1	S
Ceftriaxone	16	I
Cefepime	<=1	S
Aztreonam	4	S
Imipenem	<=1	S
Amikacin	<=2	S
Gentamicin	<=1	S
Ciprofloxacin	<=0.25	S
Piperacillin and/or Tazobactam	<=4	S
Nitrofurantoin	128	R
Levofloxacin	0.5	S

MIC, Minimal inhibitory concentration; R, Resistant; S, Sensitive.

## Conclusion

This is a case report from Asia showing that peritonitis can be caused by *E. americana. E. americana* is rarely isolated, especially as the pathogen for peritonitis. Our case report may add to the causes of peritonitis in the hospital environment. Since this organism can survive in water with simple nutritional provision, hospitals’ in-house sources of water, including air conditioning units, ice baths, wound cleaning devices and catheterization, can be the sources of infection.

Peritonitis is mainly caused by the disruption of the peritoneum, simply by letting micro-organisms into the peritoneal cavity, such as under the condition of CAPD. The most common isolates include cutaneous species, such as *Staphylococcus aureus*, and coagulase-negative staphylococci. Thus, the empiric choice of antibiotics often targets *S. aureus* (vancomycin), whereas in our case it should be noted that *E. americana* can also be a cause of peritonitis and its corresponding therapy should be taken into consideration.

## Consent

Written informed consent was obtained from the patient for publication of this case report and accompanying images. A copy of the written consent is available for review by the Editor-in-Chief of this journal.

## Abbreviations

CAPD: Continuous ambulatory peritoneal dialysis; PD: Peritoneal dialysis; MIC: Minimum inhibitory concentration.

## Competing interests

The authors declare that they have no competing interests.

## Authors’ contributions

LL analyzed the case, performed the microbiological examination and was a major contributor in writing the manuscript. JXS interpreted the test results of our patient. JMT performed the laboratory tests. ZZX organized the relevant tests and analyzed our patient’s results. All authors read and approved the final manuscript.
